# Halofuginone Synergistically Enhances Anti-Proliferation of Rapamycin in T Cells and Reduces Cytotoxicity of Cyclosporine in Cultured Renal Tubular Epithelial Cells

**DOI:** 10.1371/journal.pone.0144735

**Published:** 2015-12-15

**Authors:** Tony L. H. Chu, Qiunong Guan, Christopher Y. C. Nguan, Caigan Du

**Affiliations:** 1 Department of Urologic Sciences, University of British Columbia, Vancouver, British Columbia, Canada; 2 Immunity and Infection Research Centre, Vancouver Coastal Health Research Institute, Vancouver, British Columbia, Canada; Fraunhofer Research Institution of Marine Biotechnology, GERMANY

## Abstract

Both rapamycin (RAPA) and cyclosporin A (CsA) are commonly used for immunosuppression, however their adverse side effects limit their application. Thus, it is of interest to develop novel means to enhance or preserve the immunosuppressive activity of RAPA or CsA while reducing their toxicity. Halofuginone (HF) has been recently tested as a potential immunosuppressant. This study investigated the interaction of HF with RAPA or with CsA in cell cultures. Cell proliferation in cultures was determined using methylthiazol tetrazolium assay, and cell apoptosis assessed by flow cytometric analysis and Western blot. The drug-drug interaction was determined according to Loewe’s equation or Bliss independence. Here, we showed that addition of HF to anti-CD 3 antibody-stimulated splenocyte cultures induced synergistic suppression of T cell proliferation in the presence of RAPA, indicated by an interaction index (γ) value of < 1.0 between HF and RAPA, but not in those with CsA. The synergistic interaction of RAPA with HF in the suppression of T cell proliferation was also seen in a mixed lymphocyte reaction and Jurkat T cell growth, and was positively correlated with an increase in cell apoptosis, but not with proline depletion. In cultured kidney tubular epithelial cells, HF attenuated the cytotoxicity of CsA. In conclusion, these data indicate that HF synergistically enhances anti-T cell proliferation of RAPA and reduces the nephrotoxicity of CsA *in vitro*, suggesting the potential use of HF for enhancing anti-T cell proliferation of RAPA and reducing CsA-mediated nephrotoxicity.

## Introduction

In general, the immunosuppression protocol for transplant rejection in patients involves combining several immunosuppressive drugs that targets different phases of T cell activity. The first step generally involves induction therapy, which uses an antibody to block T cell recognition or activation at time of transplantation. Then, immunosuppression is maintained through a combination of drugs, such as calcineurin inhibitors (CNI), steroids, and proliferation signal inhibitors (PSI), in order to protect the transplanted organ from T cell responses [1, 2]. Cyclosporine (CsA) is a CNI that binds to cyclophilin. This process results in the inactivation of calcineurin and nuclear factor of activated T cell (NF-AT) transcription factor, which in turn reduces cytokine [i.e. interleukin (IL)-2] production in T cells [3, 4]. Rapamycin (RAPA) is a PSI that binds to FK506-binding protein (FKBP)-12-rapamycin-associated protein 1 (FRAP1), also known as mammalian target of rapamycin (mTOR), which halts cell cycle progression from G1 to S phase of T cells in the response to cytokine (e.g. IL-2, IL-4, and IL-15) stimulation [4, 5]. However, the use of these two drugs is associated with many adverse effects in patients. For example, prolonged use of CNI, such as CsA, may result in renal toxicity, renal dysfunction and eventual renal failure in both transplant recipients and patients with autoimmune diseases [6, 7], whereas RAPA therapy is complicated by hyperlipidaemia, myelosuppression, impaired wound healing, proteinuria, edema, pneumonitis and thrombotic microangiopathy [8]. Hence, there is an unmet need to develop a therapeutic strategy or adjuvant therapy to reduce the toxicity of agents such as CNI or RAPA without loss of their immunosuppressive therapeutic effects.

Halofuginone (HF) is a synthetic halogenated derivative of febrifugine, a natural quinazolinone alkaloid that was first found in herbal *Dichroa febrifuga* (Chang Shan) [9], and has been used for treating parasite infection in veterinary medicine [10–14]. Recently, the immunosuppressant properties of HF have been reported, and this compound has been shown to inhibit T cell proliferation [15], human Th 17 differentiation [16] and cytokine production in activated T cells [17]. In preclinical models, treatment with HF reduces the severity of experimental autoimmune encephalomyelitis, a mouse model of multiple sclerosis [16], and delayed-type hypersensitivity (DTH) responses [17]. All of these studies show promises of using HF as a potential adjuvant to CsA or RAPA in the immunosuppression protocol. However, the drug-drug interactions of HF with RAPA and CsA have not yet been investigated.

Several models have been used in the study of drug-drug interaction in pharmacology research, especially in the assessment of synergy [18–20], but a recent study shows that they all provide similar conclusions based on the analysis of published cytotoxicity data of combinations of two anti-folate agents–AG2034 and folic acid [21]. The interactions between these two drugs depend on folic acid levels–at higher levels, the synergistic interactions are more universal, while at the lower levels, the synergy is still present but less extensive [21]. Since similar conclusion can be drawn regardless of model the drug-drug interaction is based on, we assessed the interaction of HF with RAPA or with CsA using one of these models—Loewe additivity. Loewe additivity is the concept that two drugs act on a target through a similar mechanism, and a combination or interaction index is developed to denote whether these two drugs interact with each other. The three types of interaction index are antagonism (negative interaction), additive (no interaction) and synergy (positive interaction) [20, 22]. In the present study, drug-drug interaction of HF with RAPA or with CsA was investigated in the suppression of T cell proliferation in both anti-CD3 antibody- and alloantigen-stimulated splenocyte cultures, and in cell proliferation in cultured human T lymphocytes (Jurkat cells), and also the effect of HF on CsA-induced cell death in cultured human proximal tubular epithelial (HK-2) cells was examined.

## Materials and Methods

### Ethics Statement

Mouse experiments were performed in accordance with the Canadian Council on Animal Care guidelines under the protocol (No: A11-0409) approved by the Animal Use Subcommittee at the University of British Columbia (Vancouver, BC, Canada).

### Animals, Cells and Reagents

Both strains of C57BL/6j and BALB/c mice (male, 10–12 weeks old) were received from breeding colonies in the animal facility at the Jack Bell Research Centre (Vancouver, BC, Canada), and all the experiments using these mice were carried out following an approved protocol as stated above.

A single cell suspension of splenocytes was prepared from the spleens of naïve mice as described previously [15]. Both HK-2 cells (an immortalized human kidney proximal tubular cell line) and Jurkat cells (an immortalized human T cell line) were purchased from the American Type Culture Collection (ATCC, Manassas, VA, USA). Cells were grown at 37°C in a humidified atmosphere of 5% CO_2_. Mouse splenocytes and Jurkat cells were grown in RPMI 1640 complete medium (Invitrogen, Burlington, ON, Canada) containing 10% fetal bovine serum (FBS) and 100 U/mL penicillin/streptomycin. HK-2 cells were grown in K1 complete culture medium as described previously [23]. HF was purchased from the Toronto Research Chemicals Inc. (Catalogue number: H102500, Toronto, ON, Canada). RAPA was from Caymen Chemical Company (Catalogue number: 13346, Ann Arbor, MI, USA). CsA was from Novartis (Catalogue number: 486205, Mississauga, ON, Canada). Anti-mouse anti-CD3 antibody was purified from anti-mouse CD3 hybridoma ascite [24].

### Preparation of TCR-Stimulated T Cells

T cell proliferation was activated by anti-CD3 antibody in splenocyte cultures, or by alloantigens in a one-way mixed lymphocyte reaction (MLR). In anti-CD3 antibody stimulated splenocyte cultures, T cell proliferation of naïve splenocytes (2 ×10^5^ cells in 100 μL of RPMI complete medium per well in 96-well microplates) was stimulated by incubating with anti-CD3 antibody (2 μg/mL), and was measured after 18, 36 or 48 hrs. Non-stimulated splenocyte cultures were used as a negative control for T cell proliferation in the anti-CD3 antibody-stimulated cultures. The MLR was a mixed culture consisting of splenocytes (2 × 10^5^/well) from BALB/c mice (H-2^d^) and mitomycin C (Sigma-Aldrich Canada, Oakville, ON, Canada) pre-treated splenocytes (1 × 10^5^/well) from C57BL6/j mice (H-2^b^) in 96-well U-bottom microculture plates (Corning Inc., Corning, NY). The splenocytes from C57BL6/j mice, used as allogeneic stimulators, were prepared by pre-treating the cells (1 × 10^6^ cells/ml) with 50 μg/ml mitomycin C at 37°C for 30 min, followed by extensively washing with PBS. The controls for basal levels of responder proliferation (splenocytes from BALB/c mice) were the cultures without stimulator cells. Cultures were maintained in RPMI 1640 complete medium for 48 hrs at 37°C in 5% CO_2_. The T cell proliferation or viability, both in anti-CD3 antibody stimulated cultures and MLR, was quantitatively measured using methylthiazol tetrazolium (MTT) assay as described below.

### MTT Assay

Cell proliferation, or an increase in viable cell numbers in cell cultures, was measured using the MTT assay routinely in the laboratory. In brief, 10 μL of 0.5 mg/mL of 3-(4,5-dimethylthiazol-2-yl)-2,5-diphenyltetrazolium bromide (MTT, Sigma-Aldrich Canada) was added to each well (100 μL of cell culture) and incubated at 37°C for 4 hrs. The formazan crystals in viable cells were then dissolved in 100 μL/well of dimethyl sulfoxide (DMSO, Sigma-Aldrich Canada). The absorbance of the color in each well, indicating the viability of cells, was quantified at a 560 nm wavelength using an ELx808 Ultra Microplate Reader (BioTek, Winooski, VT, USA). The percent inhibition of cell proliferation/viability in drug-treated cultures against nondrug-treated control was calculated as follows: Inhibition (%) = (Control—Drug-treated)/Control ×100%. The IC_50_ or IC_70_ was determined from the mean concentration of an inhibitor that decreased cell growth by 50% or 70% from several separate experiments, respectively.

### Trypan Blue Exclusion Assay

Trypan blue exclusion assay was used to confirm the cell proliferation measured by the MTT assay, in which viable cells were identified by negatively staining of trypan blue, a cell membrane impermeable dye. In brief, after 48 hrs of drug treatment, all the cells including adhesive cells were detached with trypsin-EDTA solution (Sigma-Aldrich Canada), followed by staining with trypan blue solution. The numbers of viable cells (trypan blue negative) were counted using a TC10™ automated cell counter (Bio-Rad Laboratories Canada, Mississauga, ON, Canada), and in each sample were presented as an average of at least three determinants. The percent inhibition of cell proliferation/viability in drug-treated cultures compared to nondrug-treated control was calculated using the same equation as described above.

### Determination of Drug-Drug Interaction

The drug-drug interaction of HF with RAPA or with CsA was determined mainly using Loewe’s equation [20, 22]. The interaction of two drugs was calculated using an interaction index (γ): γ = (*d*_*1*_/*D*_*1*_) + (*d*_*2*_/*D*_*2*_), where *D*_*1*_ and *D*_*2*_ were the concentrations of drug 1 alone and drug 2 alone that resulted in a given percentage of inhibition (i.e. 50% or 70%), respectively, and *d*_*1*_ and *d*_*2*_ were the concentrations of drug 1 and drug 2 in the combination that yielded the same inhibition (50% or 70%). The drug-drug interaction was additive if γ = 1, super-additive (synergistic) if γ < 1, or sub-additive (antagonistic) if γ > 1.

Also, the drug-drug interaction between HF and RAPA was confirmed by using a Bliss independence model, in which the theoretical combined inhibition (τ) was calculated as follows: τ = *E*_*1*_ + (100%—*E*_*1*_) *× E*_*2*_, where *E*_*1*_ and *E*_*2*_ were the percent inhibition of drug 1 alone and drug 2 alone, respectively. The actual combination inhibition (α) was measured in the cultures treated with the same concentrations of both drug 1 and drug 2. The drug-drug interaction was additive if τ = α, super-additive (synergistic) if τ < α, or sub-additive (antagonistic) if τ > α.

### Determination of Cell Apoptosis by a Flow Cytometry

Apoptosis both in T cell and HK-2 cultures was quantitatively determined by fluorescence-activated cell sorter (FACS) analysis with double stain with annexin-V conjugated with phycoerythrin (annexin-V-PE) and 7-amino-actinomycin D (7-AAD) following the manufacturer’s protocol (BD Biosciences, Mississauga, ON, Canada). The background of this assay is that 7-AAD is a cell membrane impermeable dye and stains DNA only when the structure of the cell membrane (i.e. plasma membrane) is disrupted, such as in necrotic or late apoptotic cells, while annexin-V is a protein specifically binding to phosphatidylserine (PS) that is only localized on the intracellular (cytosolic) face of plasma membrane in viable cells–meaning negative stain of viable cells by annexin-V-PE. When cells start apoptosis, the PS is turned over to the extracellular face of the plasma membrane, resulting in positive stain with the annexin-V-PE. Briefly, cells were stained with annexin-V-PE and 7-AAD for 15 min in the dark, and cell apoptosis and necrosis were detected by using a flow cytometry and further quantified using FlowJo software (Tree Star Inc., Ashland, OR, USA). In a FACS graph, upper left quadrant contained single-7-AAD positive cells (necrosis), upper right quadrant double-annexin V/7-AAD positive cells (late apoptosis), lower left quadrant double-annexin V/7-AAD negative cells (viable cells), and lower right quadrant single-annexin V positive cells (early apoptosis).

### Western Blot Analysis

The cleaved form of poly ADP ribose polymerase (PARP) was examined as a biomarker of apoptosis using Western blot analysis as described previously [15]. In brief, splenocytes in 6-well plates were stimulated with 2 μg/mL of anti-CD3 antibody, and were treated with 2.5 nM HF in the absence or presence of 1 nM RAPA for 48 hrs. The total protein extract was prepared by a brief sonication of cell suspension in RIPA buffer (50 mM Tris-HCl, pH 8.0, 150 mM NaCl, 1% Nonidet P-40, 0.5% sodium deoxycholate, and 0.1% SDS) containing protease inhibitor cocktail (Roche, Mannheim, Germany), and fractioned by 15% SDS-polyacrylamide gel electrophrosis (SDS-PAGE). After transferring to nitrocellulose membranes (Bio-Rad Lab, Hercules, CA, USA), the cleaved PARP bands were specifically identified using rabbit anti-cleaved PARP (clone D64E10, Cell Signaling Technology, Danvers, MA, USA) along with horseradish peroxidase (HRP)-conjugated secondary antibodies. The blots of cleaved PARP were re-probed with anti-β-actin IgG antibody (Sigma-Aldrich Canada) to confirm equal protein loading in each sample. The expression levels were measured using a densitometry, in which the image of Western blot was captured with 3 different exposure times, and the level of a target protein (band) was measured based on its size (length x width) and dark intensity. Data were presented as a ratio unit (RU) of PARP to β-actin on the same image of the blots.

### Statistical Analysis

Data were presented as mean ± standard derivation (SD) of separate experiments (at least three experiments if results were consistent). Statistical analysis of difference between groups was performed by *t*-test (two-tailed distribution) or analysis of variance (ANOVA) as indicated in the text. A p value of < 0.05 was considered statistically significant.

## Results

### HF synergistically enhances RAPA in the suppression of T cell proliferation

**Fig 1:**
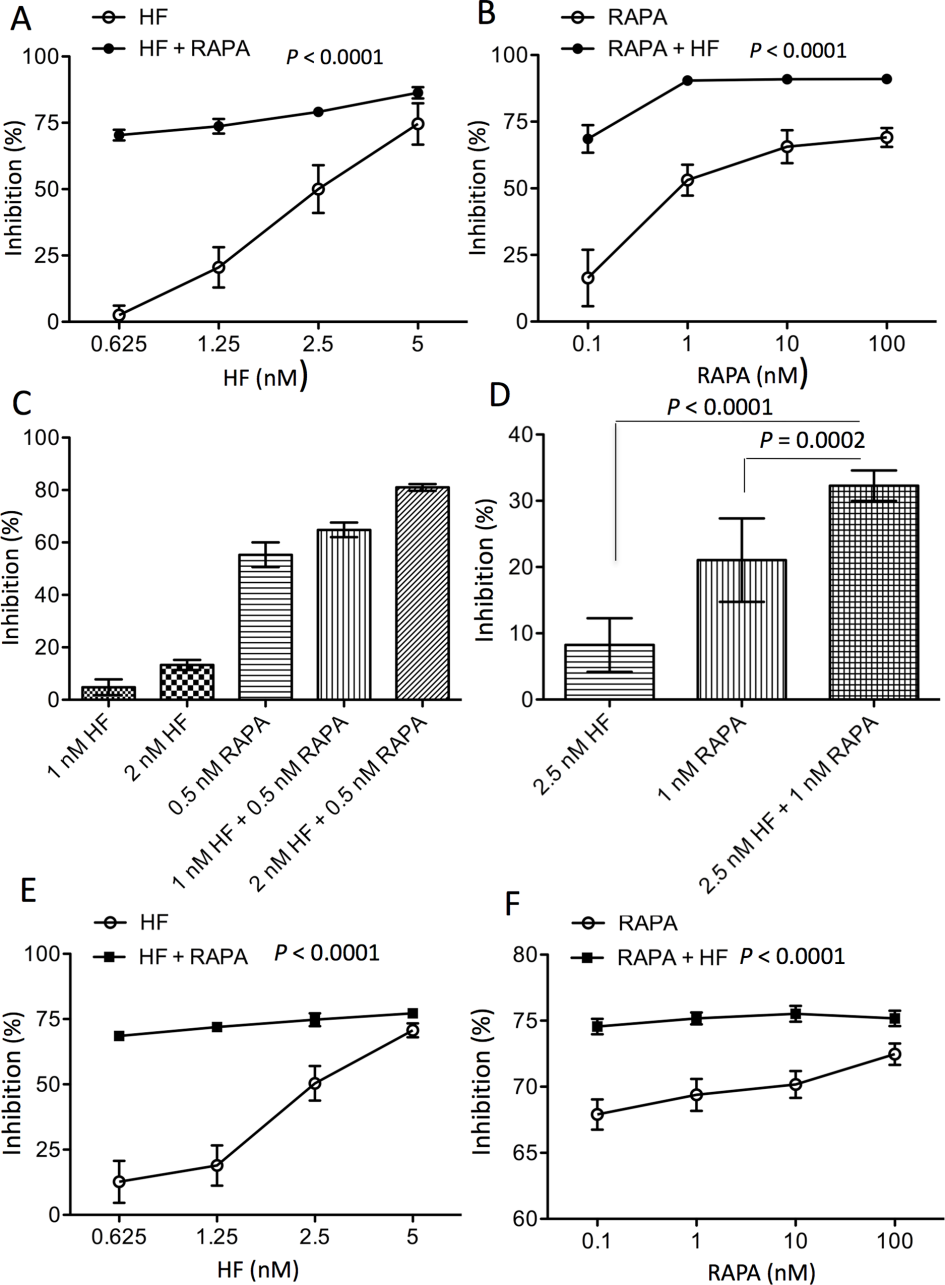
HF synergistically interacts with RAPA in the suppression of TCR stimulated T cell proliferation. T cell proliferation in response to the stimulation of anti-CD3 antibody binding to TCR complex was measured by using MTT assay or trypan blue exclusion. (A) Anti-CD3 antibody-stimulated splenocytes were treated with HF alone or in combination with 1 nM of RAPA for 48 hrs. (B) Anti-CD3 antibody-stimulated splenocytes were treated with RAPA alone or in combination with 2.5 nM of HF for 48 hrs. Data are presented as mean ± SD of five separate experiments, and were statistically analyzed using two-way ANOVA. P < 0.0001 (HF *vs*. HF + RAPA); P < 0.0001 (RAPA *vs*. RAPA + HF). γ = 0.1905 based on IC_70_ values in these two experiments. (C) Anti-CD3 antibody-stimulated splenocytes were treated with HF (1 or 2 nM) or RAPA (0.5 nM) alone or in combination with both drugs for 48 hrs, followed by counting viable cells using trypan blue exclusion assay. Data are presented as mean ± SD of three experiments. The Bliss independence: τ < α. (D) The splenocyte cultures were treated with HF alone, RAPA alone or both HF and RAPA for 18 hrs, and the cell growth was determined by using MTT assay. The difference of inhibition between groups was analyzed using two-tailed *t*-test. Data are presented as mean ± SD of five experiments. The Bliss independence: τ < α. (E) The splenocyte cultures were treated with HF alone or in combination with 1 nM RAPA for 36 hrs. (F) The splenocyte cultures were treated with RAPA alone or in combination with 2.5 nM HF for 36 hrs. The cell growth was determined using MTT assay. Data are presented as mean ± SD of five experiments. The difference of drug inhibitions between groups was analyzed using two-way ANOVA. P < 0.0001 (HF *vs*. HF + RAPA); P < 0.0001 (RAPA *vs*. RAPA + HF). γ = 0.201 based on IC_70_ values in these two experiments.

RAPA has been used as an anti-lymphocyte (T and B cells) agent for preventing organ transplant rejection [25]. To determine whether HF could improve its anti-T cell proliferative activity, the interaction between HF and RAPA on T cell growth was examined in various cultures. In anti-CD3 antibody-stimulated splenocytes, HF alone inhibited T cell proliferation (IC_70_ = 3.2 nM), and in combination with 1 nM of RAPA the inhibition was significantly increased to 74.0 ± 2.42% in cultures with 0.625 nM of HF or 86.35 ± 2.11% with 5 nM of HF (IC_70_ = 0.6 nM, n = 5) (HF alone *vs*. HF + RAPA: P < 0.0001, two-way ANOVA) (Fig 1A). Similarly, RAPA alone inhibited T cell proliferation in a dose-dependent manner (IC_70_ = 100 nM) (Fig 1 B), and the combination with 2.5 nM of HF also significantly increased the inhibition to 68.58 ± 5.17% in cultures with 0.1 nM of RAPA or 91.04 ± 0.42% with 100 nM of RAPA (IC_70_ = 0.3 nM, n = 5) (RAPA alone *vs*. RAPA + HF: P < 0.0001, two-way ANOVA)(Fig 1B). The combination index γ value of RAPA and HF interaction in the anti-CD3 antibody-stimulated splenocyte cultures was equal to 0.1905. The T cell proliferation in these splenocyte cultures was also measured by using trypan blue exclusion assay (Fig 1C), and the interaction between HF and RAPA was calculated using the Bliss independence model. As shown in Fig 1C, addition of 0.5 nM RAPA inhibited 55.3 ± 4.7% of cell growth, while 1 or 2 nM of HF alone resulted in 4.8 ± 3% or 13.3 ± 1.9%. The theoretical combination (τ) of 0.5 nM RAPA with 1 or 2 nM HF was 57.45% or 61.25%, which was significantly less than their actual combination inhibition (α) (64.8 ± 2.8% or 81 ± 1.3%), suggesting the synergistic interaction of these two drugs in trypan blue exclusion assay.

To further confirm the synergistic interaction between HF and RAPA in the suppression of T cell proliferation in the response to anti-CD3 antibody stimulation, the interactions of RAPA with HF was examined in the cultures at earlier time points (18 and 36 hrs) even when the cell proliferation was not highly stimulated as compared to that at 48 h. Because cell proliferation was not significantly induced at 18 hrs, the drug-drug interaction between RAPA and HF was examined in the splenocyte cultures in absence (untreated control) or presence of 2.5 nM HF, 1 nM RAPA or both 2.5 nM HF and 1 nM RAPA. As shown in Fig 1D, the inhibition (%) of cell growth by each drug treatment was calculated as compared to that in untreated control cultures, and it was 8.24 ± 4.04% (n = 5) by HF alone, 21.05 ± 6.29% (n = 5) by RAPA alone, or 32.29 ± 2.30% (n = 5) by both RAPA and HF. According to the Bliss independence model, the theoretical sum of inhibition of 1 nM RAPA and 2.5 nM HF combination was equal to 27.56% [21.05% + (100–21.05) × 8.24%], that was much less than 32.29 ± 2.30%, the actual value from the experiment, suggesting their synergistic interaction at this time point. The HF-RAPA interaction was also investigated at 36 hrs in the same cultures as we did at 48 hrs as described above. HF alone inhibited 70.72 ± 2.66% of cell proliferation (IC_70_) at 5 nM, and in combination with 1 nM RAPA, the concentration of HF for IC_70_ was significantly decreased to 1 nM, as evidenced by 68.55 ± 0.85% in the combination of 1 nM RAPA with 0.625 nM HF or 71.98 ± 0.91% in 1 nM RAPA with 1.25 nM HF (HF alone *vs*. HF + RAPA: P ˂ 0.0001, two-way ANOVA, n = 5) (Fig 1E). While 10 nM RAPA alone resulted in 70.18 ± 1.02% of inhibition (IC_70_ = 10 nM), and in combination with 2.5 nM HF, the inhibition was increased to 75.53 ± 0.6%. The combination of 2.5 nM HF with 0.1 nM RAPA inhibited 74.56 ± 0.58%, indicating that in the presence of 2.5 nM HF, IC_70_ of RAPA was the less than 0.1 nM (RAPA alone *vs*. RAPA + HF: P ˂ 0.0001, two-way ANOVA, n = 5) (Fig 1F). The γ value of RAPA and HF interaction using Loewe’s equation in these splenocyte cultures was equal to 0.201. Taken together, all these data suggest the synergistic interaction of these two drugs not only at 48 hrs but also at early time points (18–36 hrs).

In addition, the interaction between RAPA and HF in the suppression of T cell proliferation was further examined in both MLR and Jurkat cell (immortalized human T cells) cultures. As shown in Table 1, the IC_70_ values of RAPA and/or HF in alloantigen-stimulated MLR were similar to those in anti-CD3 antibody-stimulated naïve splenocytes as indicated in Fig 1, but were much lower than those in Jurkat cell cultures. Again, the interaction of these two drugs was synergistic in the suppression of cell proliferation in both cultures (γ = 0.42 in MLR; γ = 0.59 in Jurkat cell cultures). Taken together, all these data suggest the synergistic interaction of these two agents in the suppression of T cell proliferation in the response to not only TCR stimulation (anti-CD3 antibody and alloantigens) but also to other mitogens (Jurkat cell cultures).

**Table 1:** The interaction of RAPA with HF in the suppression of cell growth in T cell cultures.

Cell culture	IC_70_ of HF alone	IC_70_ of RAPA alone	IC_70_ of HF in combination with RAPA	IC_70_ of RAPA in combination with HF	γ value
Splenocytes in MLR	3.5 ± 0.02 nM	10.5 ± 0.85 nM	1.33 ± 0.01 nM	0.4 ± 0.001 nM	0.42
Jurkat cells	271 ± 13.04 nM	86.8 ± 9.5 μM	159.6 ± 4.52 nM	200 ± 3.06 nM	0.59

One-way MLR was described in the Materials and Methods. Jurkat cells (1 × 10^4^ cells/well/100 μL) were grown in 96-well plates. Cell proliferation of these cultures was determined using MTT assay after 48 h of incubation. Both cell cultures were treated with different concentrations of HF and/or RAPA in order to reach 70% of cell growth inhibition (IC_70_). The values of IC_70_ in each group were presented as mean ± SD of three separate experiments (n = 3), and the interaction index γ was calculated using Loewe’ equation.

### No effect of RAPA on HF-induced proline-depletion

**Fig 2:**
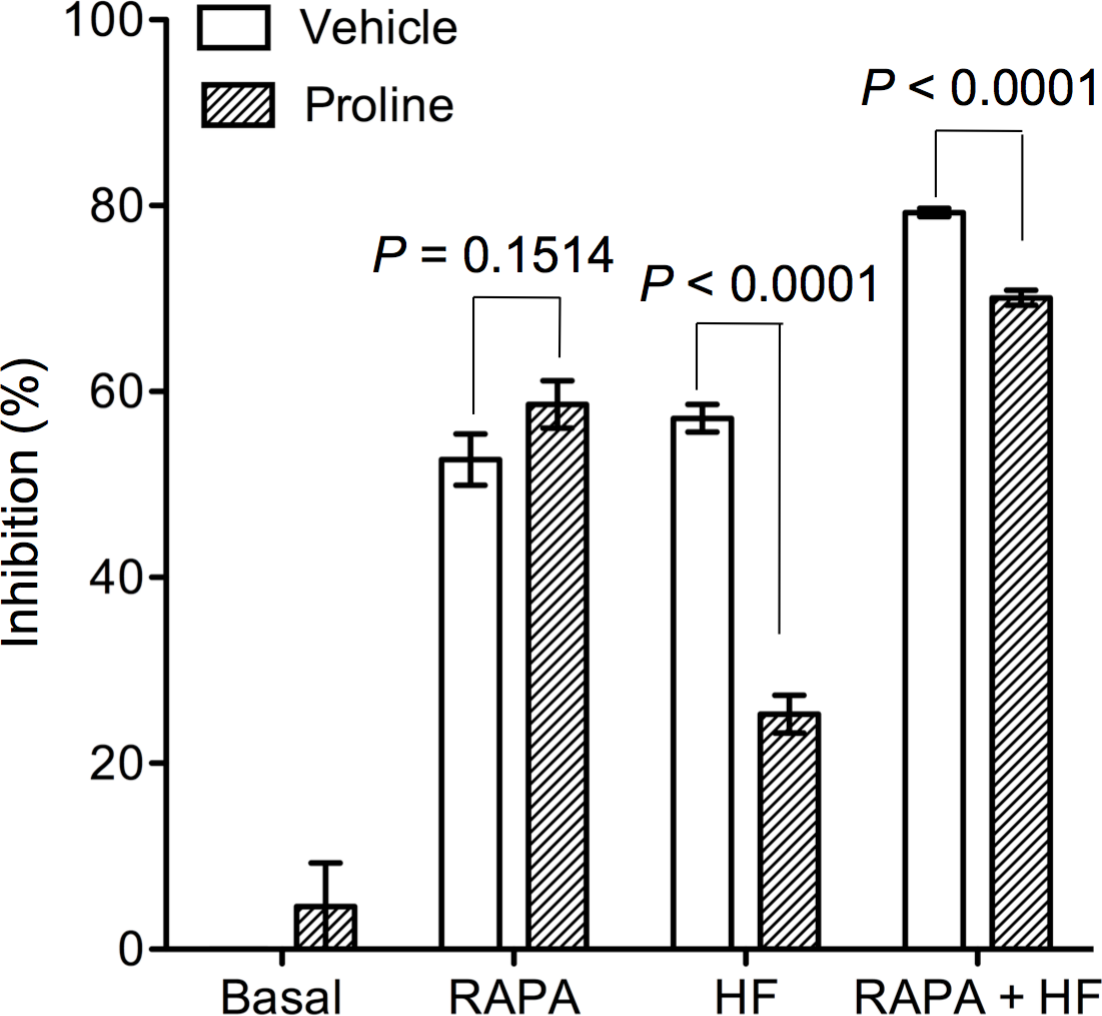
Excess proline does not interfere synergistic interaction of RAPA with HF in the suppression of T cell proliferation. Anti-CD3 antibody-stimulated naïve splenocytes were treated with HF (2.5 nM) alone, RAPA (1 nM) alone or a mixture of RAPA (1 nM) and HF (2.5 nM) in the presence of 1 mM proline or vehicle for 48 hrs. T cell proliferation was measured by using MTT assay. Data are presented as the means ± SD of three separate experiments, and were statistically compared between vehicle and proline-treated samples in each group by two-tailed *t*-test.

HF suppresses T cell proliferation by induction of intracellular proline depletion as demonstrated previously [15], while RAPA acts through inhibition of mTOR signalling [26]. To understand the mechanism by which RAPA and HF exerted synergistic interaction in the suppression of T cell proliferation, the effect of RAPA on HF-activated proline-depletion pathway was investigated. Addition of 1 mM proline completely blocked HF (1.25–5 nM) inhibition against T cell proliferation [15], but did not attenuate the anti-T cell proliferation of RAPA at the concentration of 0.1 to 100 nM in anti-CD3 antibody-stimulated splenocyte cultures (data not shown). Similarly, as shown in Fig 2 addition of proline (1 mM) did not affect RAPA (1 nM)-mediated proliferation inhibition as indicated by no difference between 58.62 ± 5.68% of inhibition by RAPA in the presence of additional proline and 52.68 ± 6.19% in the absence of additional proline (P = 0.1514, n = 5), but it reversed the inhibition of cell growth from 57.12 ± 3.33% to 25.29 ± 4.57% (approximately 57% of recovery) in HF (2.5 nM)-treated cultures (P ˂ 0.0001, n = 5). In the combination treatment of RAPA (1 nM) with HF (2.5 nM), the addition of 1 mM proline decreased the combination inhibition from 79.26 ± 1.0% to 70.08 ± 1.80% (P ˂ 0.0001, n = 5), or recovered 11.58% of the total inhibition that was much lower than that (∼57%) in cultures treated with HF alone, suggesting a decreased effect of proline depletion on the combination action of HF and RAPA.

### RAPA enhances HF-induced cell death

**Fig 3:**
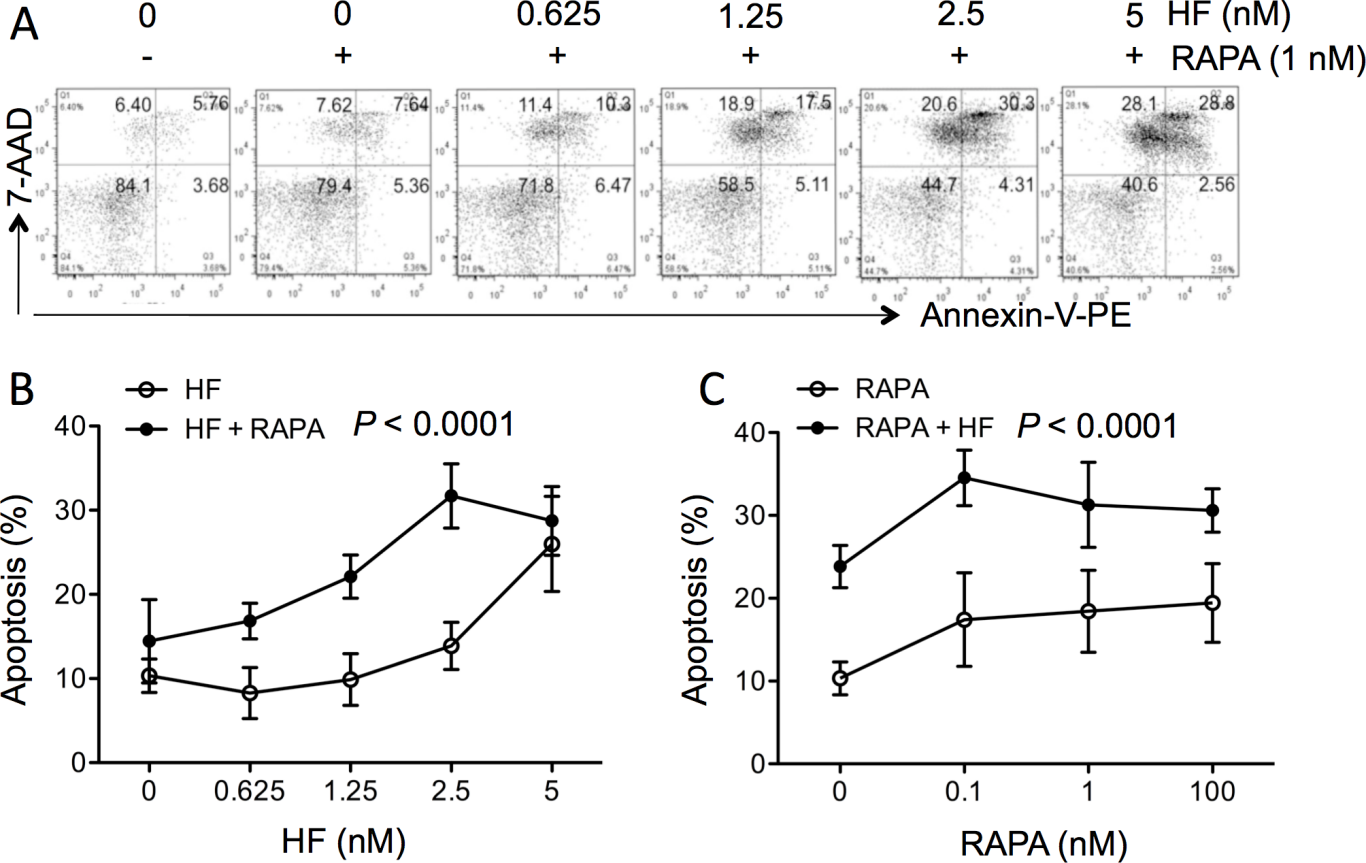
RAPA potentiates HF-induced cell death in FACS analysis. Cell death in cultured splenocytes was determined by FACS analysis with annexin-V-PE and 7-AAD staining. (A) Anti-CD3 antibody-stimulated splenocytes were treated with 1 nM of RAPA alone or in combination with various concentrations of HF for 48 hrs. Data are presented as a typical dot plot showing the percentage of annexin-V-PE and/or 7-AAD positive staining of cell populations. (B) Anti-CD3 antibody-stimulated splenocytes were treated with HF alone or in combination with 1 nM of RAPA for 48 hrs. (C) Anti-CD3 antibody-stimulated splenocytes were treated with RAPA alone or in combination with 2.5 nM of HF for 48 hrs. Apoptosis was represented by the sum of annexin-V stained cell populations (single annexin-V-PE positive cells in lower right quadrant, and double-annexin-V-PE/7-AAD positive cells in upper right quadrant). Data are presented as mean ± SD of 3–4 separate experiments, and were statistically analyzed by ANOVA. P ˂ 0.0001 (HF *vs*. HF + RAPA, n = 3); P ˂ 0.0001 (RAPA *vs*. RAPA + HF, n = 4).

**Fig 4:**
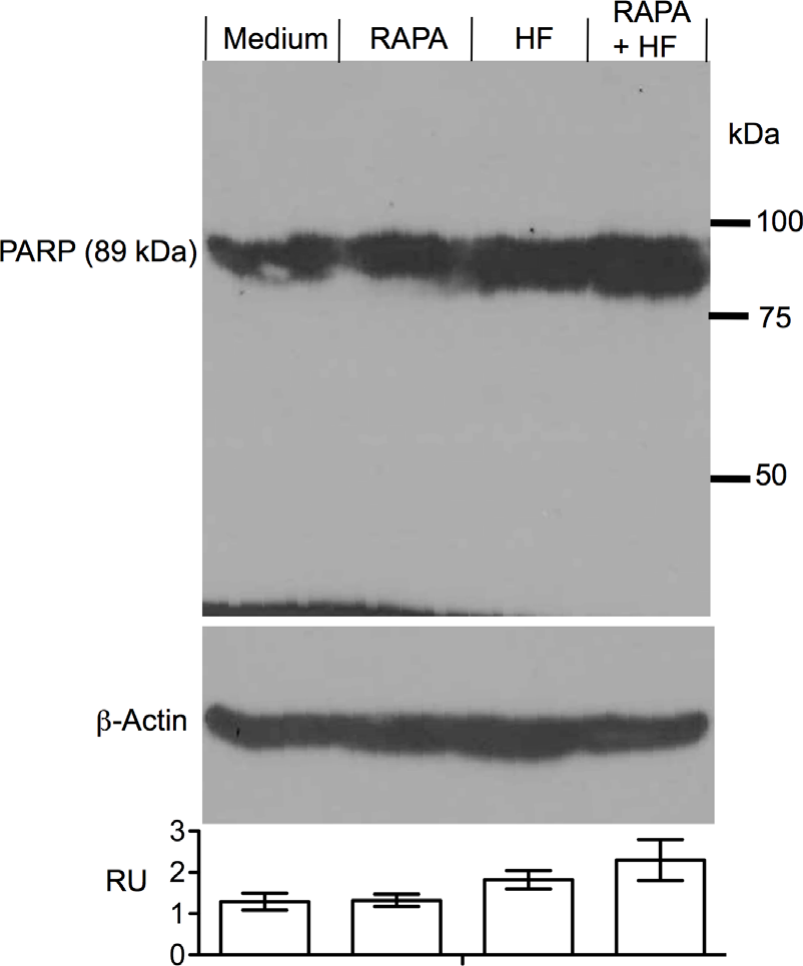
RAPA potentiates HF-induced cell death in Western blot. The cleaved form of PARP as a biomarker for cell apoptosis in protein extracts of splenocytes was analyzed by Western blot. Anti-CD3 antibody-stimulated splenocytes were treated with RAPA (1 nM) alone, HF (2.5 nM) alone or a mixture of RAPA (1 nM) and HF (2.5 nM) for 48 hrs. Equal amount of protein (approximately 250 μg) extracted from whole cell pellets was fractioned by 7% of SDS-PAGE, and the cleaved PARP protein bands was identified based on specifically binding of anti-cleaved PARP antibody, and their molecular size (cleaved PARP: 89 kDa). The protein content in each sample was confirmed by re-probing the blot with anti-β-actin IgG antibody and was measured by a densitometry. Imaging data are a representative of three separate experiments. The ratio unit (RU) of PARP band to actin band from the same sample on the same blot was presented as mean ± SD of three determinants. P ˂ 0.0001 (HF vs. HF + RAPA), P ˂ 0.0001 (RAPA vs. HF + RAPA), P = 0.0009 (HF vs. RAPA).

HF induces apoptosis or cell death in a variety of cell types including splenocytes [15, 16, 27], whereas RAPA causes cell cycle arrest in T cells [26, 28]. To examine the role of cell death in the synergistic interaction of RAPA with HF in the suppression of T cell proliferation, cell apoptosis or viability was examined using FACS analysis in anti-CD3 antibody-stimulated splenocytes treated with various concentrations of HF (0–5 nM) in the presence of 1 nM RAPA or with various concentrations of RAPA (0–100 nM) in the presence of 2.5 nM HF. As shown in Fig 3, HF alone induced cell apoptosis in a dose dependent manner (P ˂ 0.0001, one-way ANOVA, n = 3) that was similar to the results reported previously [15], and the combination of HF and RAPA (1 nM) further significantly increased the proportion of apoptotic cells (total annexin-V stained cells including both early and late apoptosis), from 8.28 ± 3.05% (0.625 nM HF alone) to 16.84 ± 2.11% (0.625 nM HF plus 1 nM RAPA), or from 13.88 ± 2.79% (2.5 nM HF alone) to 31.71 ± 3.81% (2.5 nM HF plus 1 nM RAPA) (HF alone *vs*. HF + RAPA: P ˂ 0.0001, two-way ANOVA, n = 3). A dose dependent increase in the numbers of 7-AAD stained cells (upper left quadrant in a dot plot) was also noted in these HF-treated cultures in the presence of RAPA (Fig 3A), which however was not seen in those treated with HF alone [15]. Furthermore, Fig 3C showed that RAPA alone did not affect cell apoptosis in a dose response, indicated by the inhibition from 17.43 ± 5.65% in cultures treated with 0.1 nM RAPA to 19.44 ± 4.76% with 100 nM RAPA (P = 0.0584, one-way ANOVA, n = 4), but in combination with HF the apoptosis was increased to 34.54 ± 3.34% in cultures treated with both 0.1 nM RAPA and 2.5 nM HF, and to 30.61 ± 2.61% with 100 nM RAPA and 2.5 nM HF (RAPA alone *vs*. RAPA + HF: P ˂ 0.0001, two-way ANOVA, n = 4). The potentiation of HF-induced cell death by RAPA was also confirmed using Western blot analysis (Fig 4), in which there was an increase in cleaved PARP protein levels in the cultures treated with both HF and RAPA (2.3 ± 0.08) as compared to those treated with either HF alone (1.52 ± 0.06) (HF *vs*. HF + RAPA: P ˂ 0.0001, two-tailed t-test, n = 3) or RAPA alone (1.12 ± 0.05) (RAPA *vs*. HF + RAPA: P ˂ 0.0001, two-tailed *t*-test, n = 3). Meanwhile, we failed to identify the change in the levels of uncleaved PARP proteins in these samples using Western blot analysis (data not shown).

### HF antagonizes CsA in the suppression of T cell proliferation but mitigates CsA cytotoxicity in cultured HK-2 cells

**Fig 5:**
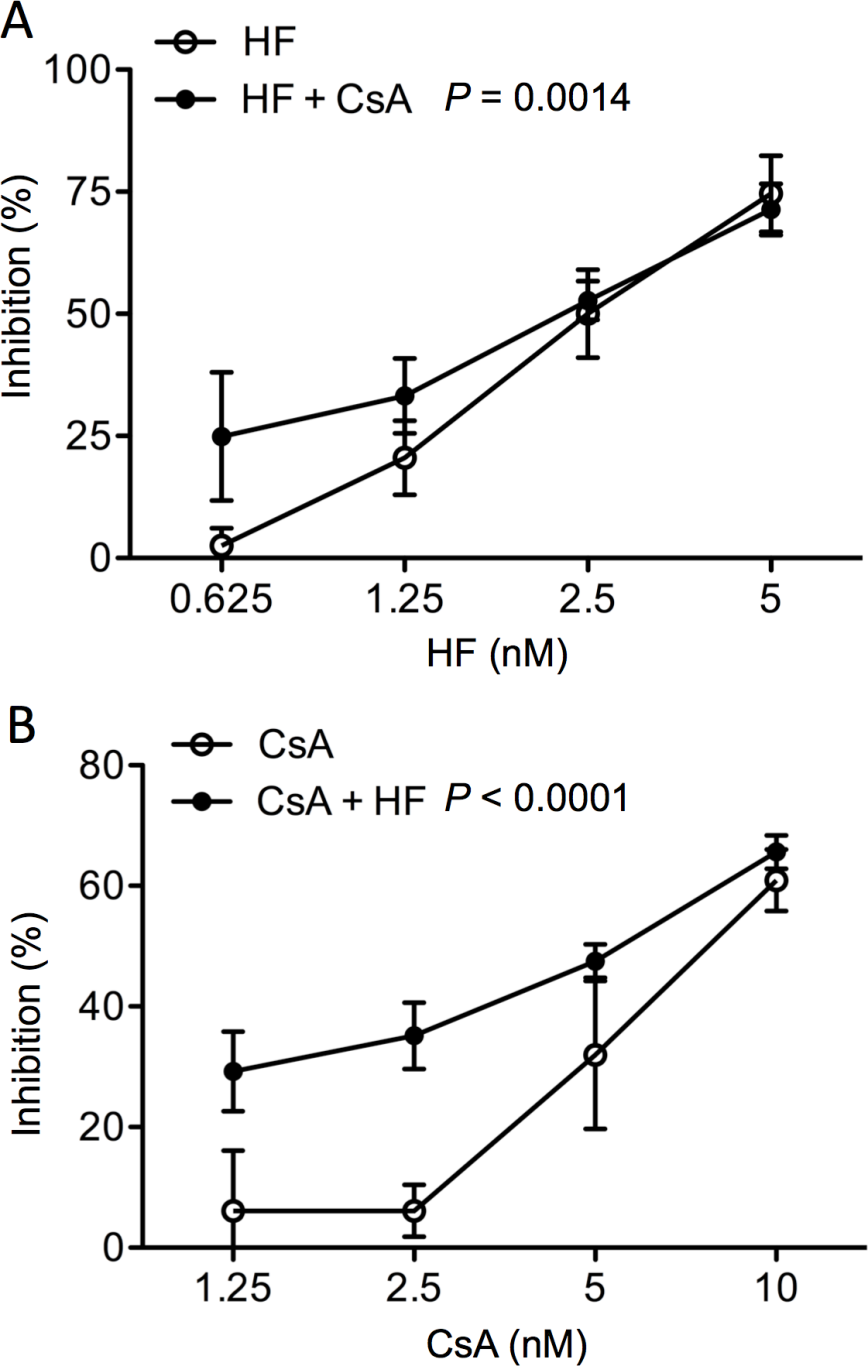
HF antagonistically interacts with CsA in the suppression of TCR stimulated T cell proliferation. T cell proliferation in response to the stimulation of anti-CD3 antibody binding to TCR complex was measured by MTT assay. (A) Anti-CD3 antibody-stimulated splenocytes were treated with HF alone or in combination with 5 nM of CsA for 48 hrs. (B) Anti-CD3 antibody-stimulated splenocytes were treated with CsA alone or in combination with 2.5 nM of HF for 48 hrs. Data are presented as mean ± SD of five separate experiments, and were statistically analyzed by ANOVA. P = 0.0014 (HF *vs*. HF + CsA); P ˂ 0.0001 (CsA *vs*. CsA + HF). γ = 1.8375 based on IC_50_ values in these two experiments.

CsA is widely used to prevent transplant rejection by suppressing the activity and growth of T cells [29], but its clinical use is largely limited by its nephrotoxicity [3, 30]. It was of interest in examining the combination effects or the interactions of HF with CsA on T cell proliferation as well as kidney cell survival. As demonstrated above (Fig 1), HF alone potently inhibited T cell proliferation in a dose dependent manner (IC_50_ = 2.5 nM; P ˂ 0.0001, one-way ANOVA, n = 5), and its inhibition was increased in the combination with 5 nM CsA as indicated by 24.9 ± 13.12% in the cultures with 0.625 nM of HF, or 71.39 ± 5.25% with 5 nM of HF (IC_50_ = 2.25 nM, n = 5) (HF alone *vs*. HF + CsA: P = 0.0014, two-way ANOVA) (Fig 5A). Whereas CsA alone inhibited the proliferation from 3.9 ± 10.3% by 1.25 nM to 54.4 ± 4.6% by 10 nM (IC_50_ = 8 nM: P ˂ 0.0001, one-way ANOVA, n = 5) (Fig 5B), and when combined with 2.5 nM HF, the inhibition of CsA at 1.25 nM was 29.23 ± 6.59%, and at 10 nM was 65.62 ± 2.77% (IC_50_ = 7.5 nM, n = 5) (CsA alone *vs*. CsA + HF: P ˂ 0.0001, two-way ANOVA)(Fig 5B). However, the γ value of these two drugs was equal to 1.8375, suggesting the antagonistic interaction in their suppression of T cell proliferation although more inhibition was seen in the combination of these two drugs.

**Fig 6:**
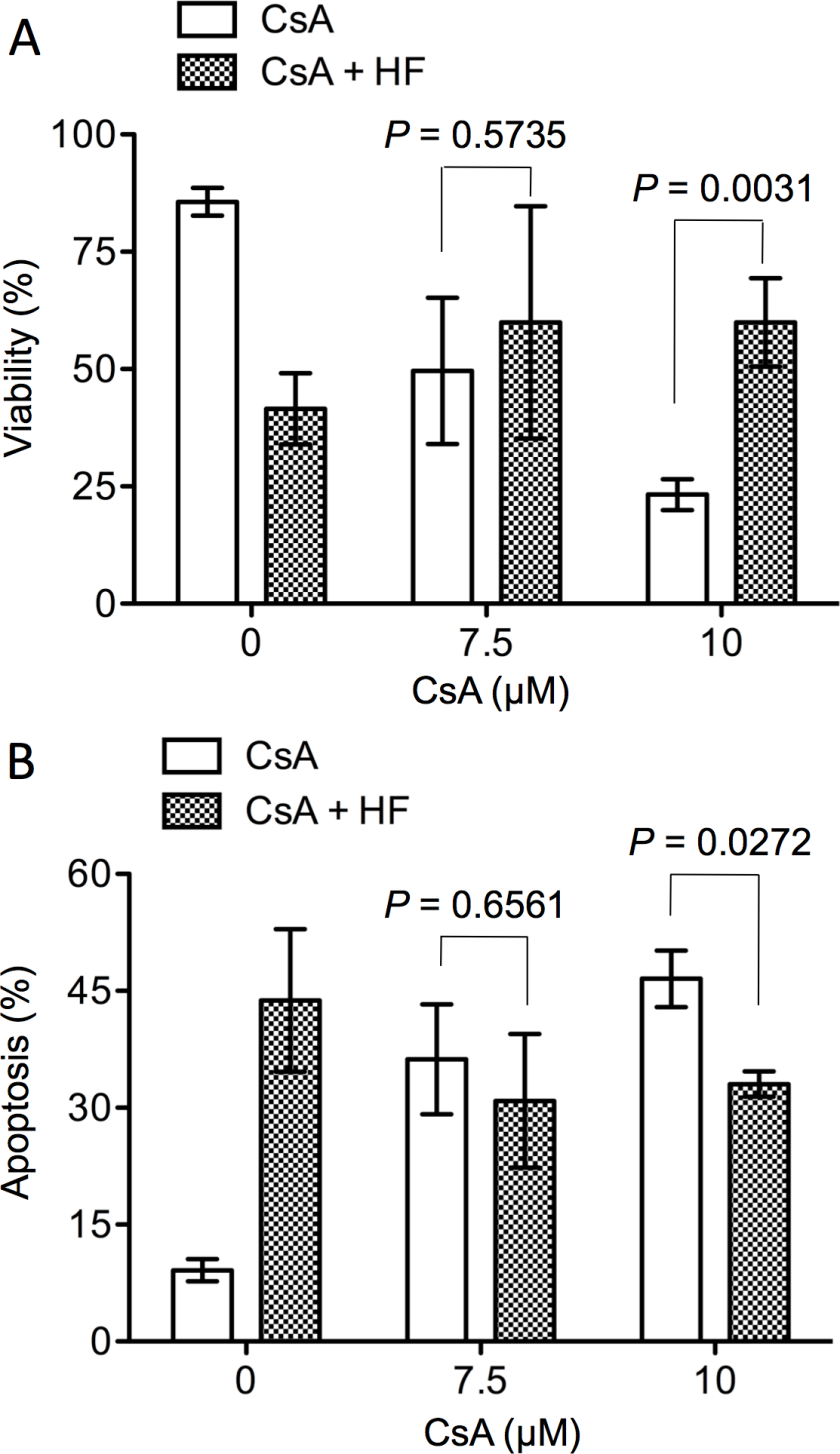
HF reduces CsA-induced cell death in cultured HK-2 cells. Cell viability or apoptosis was measured by FACS analysis with annexin-V-PE and 7-AAD staining. A monolayer of HK-2 cells was treated with CsA (0–10 μM) alone or in combination with 1 μM HF for 48 hrs. Cell viability represented the percentage of viable cells (double-annexin-V-PE/7-AAD negative cells in lower left quadrant), and apoptosis was the sum of Annexin-V stained cell populations (single annexin-V-PE positive cells in lower right quadrant, and double-annexin-V-PE/7-AAD positive cells in upper right quadrant). Data are presented as mean ± SD of three separate experiments, and were statistically compared between CsA alone and CsA with HF in each group by two-tailed *t*-test.

The nephrotoxicity of CsA was tested in an *in vitro* system—cultured HK-2 cells. In this system, a micromolar level of CsA is required to induce reproducible cytotoxicity in cultured HK-2 cells [31]. In this study, we tested the effect of HF on cell death induced by CsA at both 7.5 and 10 μM. As shown in Fig 6, CsA induced cell death in cultured HK-2 cells in a dose-dependent manner, indicated by a decrease in the proportion of viable cells from 85.63 ± 2.95% in untreated cultures to 49.63 ± 15.55% in cultures treated with 7.5 μM CsA, and 23.27 ± 3.29% with 10 μM CsA (P = 0.0005, one-way ANOVA, n = 3) (Fig 6A), or an increase in apoptosis from 9.14 ± 2.47% in untreated cultures to 36.23 ± 12.25% with 7.5 μM CsA, and 46.57 ± 6.28% with 10 μM CsA (P = 0.0033, one-way ANOVA, n = 3) (Fig 6B). However, this dose-dependent CsA-induced cell death was not seen in HK-2 cell cultures in the presence of 1 μM HF. As a matter of fact, addition of HF prevented CsA-induced cell death as the percentage of viable cells was increased from 23.27 ± 3.29% in the cultures with 10 μM CsA alone to 59.98 ± 9.44% in those with both 10 μM CsA and HF (P = 0.0098, *t*-test, n = 3) (Fig 6A), or decreased cell apoptosis from 46.57 ± 6.28% with 10 μM of CsA alone to 33.04 ± 2.82% with both CsA and HF (P = 0.0213, *t*-test, n = 3) (Fig 6B).

## Discussion

RAPA and its derivatives have been shown to be promising drugs for immunosuppressive therapy (e.g. anti-transplant rejection). And CNI, such as CsA, still remain the most widely used contemporary immunosuppressive agents in organ transplantation [7], but the prolonged use of CNI is associated with nephrotoxicity [3, 7]. Therefore, it is always of a great interest to develop an adjuvant agent that can enhance the efficacy of these drugs and/or reduce their potential toxicity. HF has been tested for its potential for immunosuppression recently [15, 16, 32]. This study shows for the first time the synergistic interaction of HF with RAPA in the suppression of T cell proliferation, but the antagonistic interaction with CsA. HF was shown to attenuate CsA-induced kidney tubular cell death *in vitro*.

MTT assay is a colorimetric method to measure the number of viable cells. It depends on the ability of viable cells to reduce the tetrazolium dye, MTT, to its insoluble formazan, and is at least as sensitive as [^3^H] thymidine uptake test in the measurement of cell proliferation in T cells [33] and keratinocytes [34]. MTT assay was mainly used to determine the cytotoxicity of HF, CsA, and RAPA in this study, and has been similarly used to determine the growth inhibitory activity of CsA and RAPA in T cells or skin keratinocytes in many previous studies [34–36]. The number of the viable cells in MTT assay also correlates with those by using flow cytometric analysis in HF-treated splenocytes [15] as well as by using trypan blue exclusion assay (Fig 1C). The effect of drugs (10 nM of HF, RAPA or CsA) on MTT activity was not found (data not shown). All these studies suggest that MTT assay can give a reliable measurement of the cytotoxicity of these drugs in different types of cells including splenocytes.

In polydrug therapy with two or more drugs, one drug is used as a base or primary drug, and the other drug(s) are added to increase the efficacy of the primary drug and/or to reduce its side effects. The synergistic interaction was found between RAPA and HF in the suppression of T cell proliferation (Fig 1, Table 1), suggesting that HF is a potential adjuvant to enhance the therapeutic effects of RAPA-based immunosuppressive protocol on T cell-mediated graft rejection. Such synergistic relationship may be attributed by their differential biological functions. HF blocks proline incorporation in an *in vitro* system [32], and inhibits proline uptake in splenocyte cultures [15]. Addition of excess proline into the culture medium reversed approximately 57% of HF-mediated inhibition of T cell proliferation (Fig 2), but only 25.7% in the cultures with both RAPA and HF, indicating that the proline depletion is not positively correlated with synergistic interaction of RAPA with HF in the suppression of cell proliferation. Our results suggest that HF-induced proline depletion may not be further enhanced or potentiated by RAPA, or the proline-depletion is not a common downstream pathway mediating the synergistic interaction of RAPA with HP in the suppression of T cell proliferation.

The blockage of proline incorporation by HF could activate amino acid starvation responses [16, 37], which consequently leads to caspase 3-dependent apoptosis as demonstrated in many previous studies [15, 16, 27], suggesting that cell apoptosis probably is the downstream pathway for HF-mediated suppression of cell growth, not only in anti-CD3 antibody-stimulated T cells but also in T cells even without antibody stimulation (i.e. non-stimulated control splenocytes, and Jurkat cells in Table 1). RAPA is a mTOR inhibitor, specifically for mTOR complex 1 (mTORC1) [26]. RAPA at different concentrations has differential biological functions; at nano-molar levels it induces cell cycle arrest via suppression of S6 kinase, a mTORC1 substrate [38], while at micro-molar levels it induces apoptosis via complete dissociation of mTORC1 and suppression of phosphorylation of eukaryotic initiation factor 4E-binding protein (4E-BP1) [26, 39–41]. Indeed, RAPA alone at 0.1–100 nM did not induce apoptosis in splenocyte cultures (Fig 3C), but significantly enhanced HF-mediated cell death as indicated by an increase in both annexin-V and 7-AAD stained cells in FACS analysis (Fig 3) or in the expression of cleaved PARP in Western blot using a specific anti-cleaved PARP monoclonal antibody (Fig 4). One has to acknowledge that we failed to identify this protein specifically using a polyclonal anti-PARP antibody in order to show both uncleaved and cleaved PARP forms in the same blot (data not shown), which may be due to the low sensitivity or specificity of this polyclonal anti-PARP antibody or to the relatively low levels of cell apoptosis in our cultures. Evidence in literature indicates that mTOR plays a role in the activation of autophagy; by inhibition of mTOR kinase activity RAPA activates the autophagy, which either protects the cells from cell death [42, 43] or induces autophagic cell death (both apoptosis and necrosis) [43]. Also, similar to our observation in T cell cultures in this study, RAPA has been reported to potentiate the cytotoxicity of paclitaxel in HeLa cells [44] and of valproic acid (a histone deacetylases inhibitor) in Burkitt leukemia/lymphoma cells [45]. Thus, it could be hypothesized that activation of autophagy by RAPA in T cells induces cell cycle arrest, but could result in autophagic cell death in the presence of pro-apoptotic HF. Therefore, in the presence of RAPA and HF both autophagic cell death and caspase 3-dependent apoptosis may be activated and mediate synergistic suppression of cell growth in T cells. Next studies are needed to prove this hypothesis.

Early studies have demonstrated that cyclophilin is a primary intracellular receptor (but may not be the only one) for CsA [46, 47], and CsA-cyclophilin complex inhibits calcineurin, resulting in inactivating specific transcription factors, such as NF-AT and NF-IL2A, and consequently reducing IL-2 production in activated T cells [48, 49]. Further studies show that there is a positive correlation of cyclophilin expression in tissue with tissue susceptibility to CsA toxicities [50], and cyclophilin mediates CsA cytotoxicity in endothelial cells [51] and along with kidney androgen-regulated protein in cultured kidney proximal tubular cells [52], suggesting that cyclophilin may also be a key mediator of CsA nephrotoxicity. It is also noticed in this study that CsA suppresses T cell proliferation at nanomolar levels, while it induces cell death in HK-2 cells at micromolar levels, suggesting that the level or biological importance of cyclophilin is different in different cell types (e.g. T cells versus HK-2 cells). As discovered in a previous study HF inhibits proline incorporation [32], which may reduce the biosynthesis of cyclophilin as well as other CsA-function mediator proteins, such as kidney androgen-regulated protein, in CsA-targeting cells. Thus, in the presence of HF, CsA exerts less effect on the suppression of T cell growth (Fig 5) or cell death in cultured HK-2 cells (Fig 6). However, this notion needs further investigation.

## Conclusions

In many cases of clinical practice, combination of several different drugs is often used to increase the effectiveness or to reduce the side effects of primary drug in the treatment of a disease or a health problem. Therefore, understanding of the drug-drug interactions is an essential step for the evaluation of the benefits to patients using polydrug therapy instead of a single drug therapy. Both CsA and RAPA are widely used for the suppression of immune response against transplanted organs or autoimmune disease, but are also associated with various side effects. The present study demonstrates for the first time that in cell culture systems, addition of HF could enhance anti-T cell proliferation of RAPA and reduce the cytotoxicity of CsA to kidney tubular cells, suggesting the potential use of HF or its derivatives to enhance the efficacy of RAPA in anti-T cell therapy or to reduce CsA nephrotoxicity, which however remain further evaluation, particularly in the *in vivo* experimental models.

## Supporting Information

S1 Fig Cell proliferation of anti-CD3 antibody-stimulated splenocytes. Naïve splenocytes of B6 mice (2 ×10^5^ cells in 100 μL of RPMI complete medium per well in 96-well microplates) were incubated in the absence (baseline control) or presence of anti-CD3 antibody (2 μg/mL). The cell growth was determined using MTT assay after different periods (18, 36 and 48 hrs) of stimulation. The fold change was calculated as a quantity change of MTT reading absorbance from the cultures without the antibody (non-stimulated control) to those stimulated with the antibody at each time point. Data are presented as mean ± standard derivation (SD) of five or six experiments. As compared to the baseline control, the cell growth (fold change) was exponentially increased from 1.64 ± 0.24 (n = 5) at 18 hrs to 11.69 ± 1.14 (n = 6) at 48 hrs (P ˂ 0.0001, one-way ANOVA) (S1 Fig). The MTT readout in these control cultures was normally very low (0.099 ± 0.0061 at 18 h, 0.0934 ± 0.0074 at 36 h, and 0.1097 ± 0.0024 at 48 h as compared to 1.2821 ± 0.1402 in cultures after 48 h of antibody stimulation), and also was reduced by 5 nM HF to almost zero.
(TIF)
