# Supplementary figures and images for: Halofuginone Synergistically Enhances Anti-Proliferation of Rapamycin in T Cells and Reduces Cytotoxicity of Cyclosporine in Cultured Renal Tubular Epithelial Cells
Source: PLoS One. 2015 Dec 15;10(12):e0144735. doi: 10.1371/journal.pone.0144735 (PMC4686009; doi:10.1371/journal.pone.0144735)

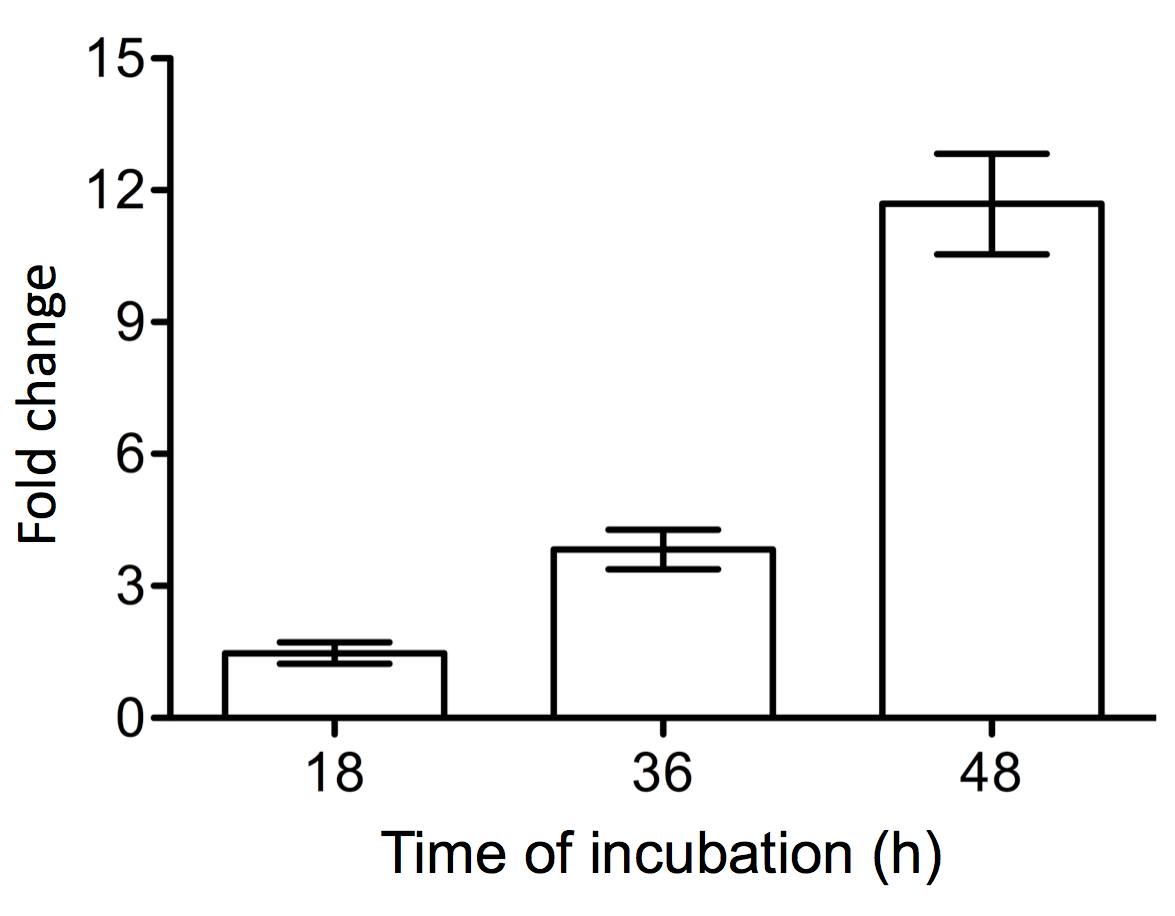

Supplement: S1 Fig — Naïve splenocytes of B6 mice (2 ×10^5^ cells in 100 μL of RPMI complete medium per well in 96-well microplates) were incubated in the absence (baseline control) or presence of anti-CD3 antibody (2 μg/mL). The cell growth was determined using MTT assay after different periods (18, 36 and 48 hrs) of stimulation. The fold change was calculated as a quantity change of MTT reading absorbance from the cultures without the antibody (non-stimulated control) to those stimulated with the antibody at each time point. Data are presented as mean ± standard derivation (SD) of five or six experiments. As compared to the baseline control, the cell growth (fold change) was exponentially increased from 1.64 ± 0.24 (n = 5) at 18 hrs to 11.69 ± 1.14 (n = 6) at 48 hrs (P ˂ 0.0001, one-way ANOVA) (S1 Fig). The MTT readout in these control cultures was normally very low (0.099 ± 0.0061 at 18 h, 0.0934 ± 0.0074 at 36 h, and 0.1097 ± 0.0024 at 48 h as compared to 1.2821 ± 0.1402 in cultures after 48 h of antibody stimulation), and also was reduced by 5 nM HF to almost zero. (TIF) [file pone.0144735.s001.tif]
